# Development and verification of a combined diagnostic model for primary Sjögren's syndrome by integrated bioinformatics analysis and machine learning

**DOI:** 10.1038/s41598-023-35864-4

**Published:** 2023-05-27

**Authors:** Kun Yang, Qi Wang, Li Wu, Qi-Chao Gao, Shan Tang

**Affiliations:** 1 School of Humanities and Social Sciences, Shanxi Medical University, Taiyuan, China; 2 School of Basic Medical Sciences, Shanxi Medical University, Taiyuan, China; 3 Shanxi Key Laboratory of Big Data for Clinical Decision Research, Taiyuan, China; 4 Key Laboratory of Cellular Physiology at Shanxi Medical University, Ministry of Education, Taiyuan, China; 5 Department of Anesthesiology, Shanxi Provincial People’s Hospital (Fifth Hospital) of Shanxi Medical University, Taiyuan, China; 6 The First Hospital of Shanxi Medical University, Taiyuan, China

**Keywords:** Computational biology and bioinformatics, Machine learning

## Abstract

Primary Sjögren’s syndrome (pSS) is a chronic, systemic autoimmune disease mostly affecting the exocrine glands. This debilitating condition is complex and specific treatments remain unavailable. There is a need for the development of novel diagnostic models for early screening. Four gene profiling datasets were downloaded from the Gene Expression Omnibus database. The ‘limma’ software package was used to identify differentially expressed genes (DEGs). A random forest-supervised classification algorithm was used to screen disease-specific genes, and three machine learning algorithms, including artificial neural networks (ANN), random forest (RF), and support vector machines (SVM), were used to build a pSS diagnostic model. The performance of the model was measured using its area under the receiver operating characteristic curve. Immune cell infiltration was investigated using the CIBERSORT algorithm. A total of 96 DEGs were identified. By utilizing a RF classifier, a set of 14 signature genes that are pivotal in transcription regulation and disease progression in pSS were identified. Through the utilization of training and testing datasets, diagnostic models for pSS were successfully designed using ANN, RF, and SVM, resulting in AUCs of 0.972, 1.00, and 0.9742, respectively. The validation set yielded AUCs of 0.766, 0.8321, and 0.8223. It was the RF model that produced the best prediction performance out of the three models tested. As a result, an early predictive model for pSS was successfully developed with high diagnostic performance, providing a valuable resource for the screening and early diagnosis of pSS.

## Introduction

Primary Sjögren’s syndrome (pSS) is a chronic, systemic autoimmune disorder^1,2^ characterized by xerostomia and xerophthalmia, which are caused by lymphocytic infiltration of the salivary and lacrimal glands^2^. In addition, the extra-glandular symptoms of pSS can also affect the joints, lungs, kidneys, liver, nervous system, and musculoskeletal system^3^. The prevalence of pSS is higher in females than in males, with the average female-to-male ratio being 9:1. Diagnosis of pSS is based on clinical signs and symptoms, which include serological tests for autoantibody biomarkers and salivary gland histopathology^4^. Owing to disease heterogeneity and its complex clinical phenotypes, the underlying pathogenesis remains unclear. Therefore, identifying biomarkers and constructing novel diagnostic models for pSS are important in understanding disease progression.

The diagnosis model has been developed using machine learning algorithms such as random forest (RF), support vector machines (SVM), and artificial neural networks (ANN). In the absence of a priori assumptions, RF analysis can identify hidden factors that distinguish between case and control groups with a high level of predictive accuracy^5^. An ANN based algorithm based on deep learning can help identify patterns and features in large volumes of data^6,7^. ANN learn to recognize patterns in data based on examples without assuming anything about the nature or interrelationships of the data. In comparison with conventional models based on polynomials, linear regression, and statistics, ANNs are competitive^8,9^. An SVM is a machine-learning algorithm that uses multivariate statistical analysis to classify and predict individuals^10^. With SVM, high-dimensional data can be effectively handled, and classification results can be obtained without overfitting^11^. To this end, the identification of reliable and efficient biomarkers that assist in early diagnosis of pSS would be of great benefit in implementing effective interventions. Li et al.^12^ identified potential biomarkers for pSS disease progression using transcriptome sequencing and clinical data by constructing a diagnostic model for pSS using circRNAs and clinical features (AUC = 0.93)^13^. Additionally, Nishikawa et al. reported that serological biomarkers may be potential therapeutic targets for pSS^14^. To date, the application of machine-learning techniques in clinical settings for diagnosis and outcome prediction has already proven successful in the context of a range of diseases^15,16^.

**Figure 1:**
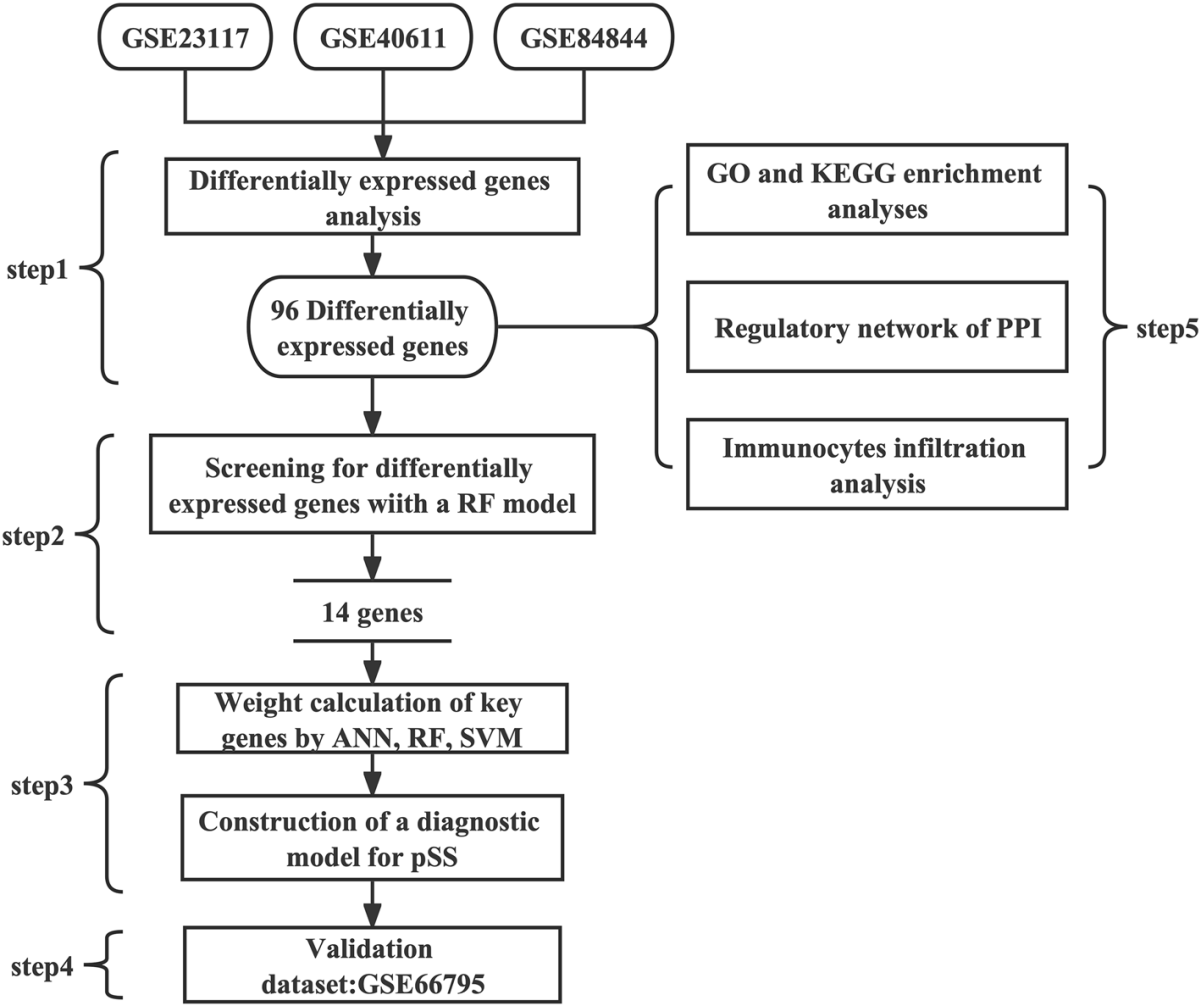
Flow-chart illustrating the study protocol.

The central idea of genomic medicine is that outcomes are improved when genetic diagnoses and genotype-individualized treatments are augmented by symptom-based diagnostics. To develop a transcriptome diagnostic model for pSS, microarray data was gathered from the Gene Expression Omnibus (GEO). Through bioinformatic analysis, we identified genes that were differentially expressed in pSS patients by comparing pSS samples with samples from patients without pSS. First, RF was used to find the genes that mattered most for classification. We developed a diagnostic model for pSS patients using three machine learning algorithms: ANN, RF, and SVM. Receiver operating characteristic (ROC) curves were used to evaluate the diagnostic performance of the chosen biomarkers. In addition, we validated the accuracy and reliability of the models by analysis using an external GEO cohort (see Fig. 1).

## Materials and methods

### Data download and processing

We downloaded microarray expression datasets from the National Center for Biotechnology Information Gene Expression Omnibus database (NCBI GEO; https://www.ncbi.nlm.nih.gov/geo/). As shown in Table 1, we searched for four sets of patients with pSS and normal controls. To create a large training cohort (GSE137684, GSE137354, and GSE34526), we used the 'ComBat' algorithm from the 'SVA' R package (version 3.46.0) to remove batch effects in different training datasets^17^. Where multiple probes mapped to the same Gene ID, the maximum mean expression value of all probes represented the gene's expression level. Probe IDs were converted to gene symbols based on the annotation of the microarray platforms. The final training dataset consisted of 57 pSS patients and 53 non-pSS samples. GSE66795 was used as the validation dataset.

**Table 1:** Source of GEO datasets.

	Training set		Testing set	
	GSE23117	GSE40611	GSE84844	GSE66795
Sample Count	15	35	60	160
pSS	10	17	30	131
Normal	5	18	30	29

### Screening for differentially expressed genes

In the training set, differentially expressed genes (DEGs) were identified using the ‘limma’ package in the ‘R’ software package (version 3.54.2) ^18^, with an adjusted P-value < 0.05 and | log2 fold-change (log2FC) |≥ 1. To create a heat map and analyze clusters of DEGs, we used the R package ‘pheatmap’. Heatmap and volcano plot visualizations of the DEGs were performed using R packages ‘pheatmap’ (version 1.0.12) and ’ggplot2’ (version 3.4.2), respectively.

### Functional enrichment analysis and construction of protein-protein interaction network

To better understand the biological significance of the DEGs, we conducted GO and KEGG enrichment analyses using the R package ’clusterProfiler' (version 4.7.1) ^19,20^. A significantly enriched pathway exhibited a *p* < 0.05 and a corrected *p* < 0.05. The STRING database (https://cn.string-db.org/) was used to analyze the network of protein-protein interactions (PPIs). The network was visualized using the ‘Cytoscape’ software package (v3.7).

### Screening for signature genes by random forest

To establish a RF model based on DEGs, the R package ‘randomForest’ was adopted (version 4.7-1.1)^21^. Signature genes were selected based on the minimum cross-validation error. We set the number of decision trees to 500 and the number of seeds to 12,345,678. Using the Gini index, signature genes in the RF model were evaluated using a gene importance score, and a score of > 1 was selected. The ‘Heatmap’ function in R was then used to cluster signature genes bidirectionally based on their expression profiles.

### Construction of the diagnostic model using machine learning

In order to eliminate batch effects in the pSS and normal groups, we converted the expression data of signature genes into ‘Gene Score’ using the min-max method. The experimental procedure was as follows: firstly, the median expression of the genes expressed in all samples was calculated. If an upregulated gene expression in a sample was greater than the median expression value of the gene, the expression was marked as 1; otherwise, it was marked as 0. Similarly, if a downregulated gene expression in a sample was greater than the median expression value of the gene, the expression was marked as 0; otherwise, it was marked as 1. Above all, the ‘Gene Score’ sheet was used for ANN analysis. The ANN model was implemented using the "neuralnet" function in R (version 1.44.2)^22^. With the neuralnet package, you can build feedforward neural networks that include one or more hidden layers^23^. A variety of popular learning algorithms are included, including backpropagation and resilient backpropagation. Additionally, learning rates and momentum can be customized. For smaller datasets, the neuralnet package provides fast and efficient performance^24^. The random seed size was set at 12,345,678. The model consisted of three types of layers: the input layers, with the ‘Gene Score’ of signature genes; the hidden layers; and the output layers, with two nodes (control/pSS). Using the expression ‘GeneExpression’ × ‘NeuralNetworkWeight’, we constructed a pSS disease diagnostic model. In addition, we also used two predictive models: RF and SVM. Based on the hub gene set, SVM classifiers were constructed using the R package e1071 (version 1.7-13). RandomForest R package (version 1.7-11) was used to train the RF classifier model. In the training and validation sets, ROC curves were generated using the ‘pROC’ package^25^ and the AUC represented the diagnostic value.

### Identification of immune cell infiltration

With the LM22 signature as a reference, CIBERSORT^26^ was used to characterize tumor-infiltrating immune cells within the pSS and normal groups in the training set. The R function ‘corrplot’ (version 0.92) was used to calculate Spearman’s correlations relating to immune cell infiltration.

## Results

### Screening of DEGs and functional enrichment analysis

**Figure 2:**
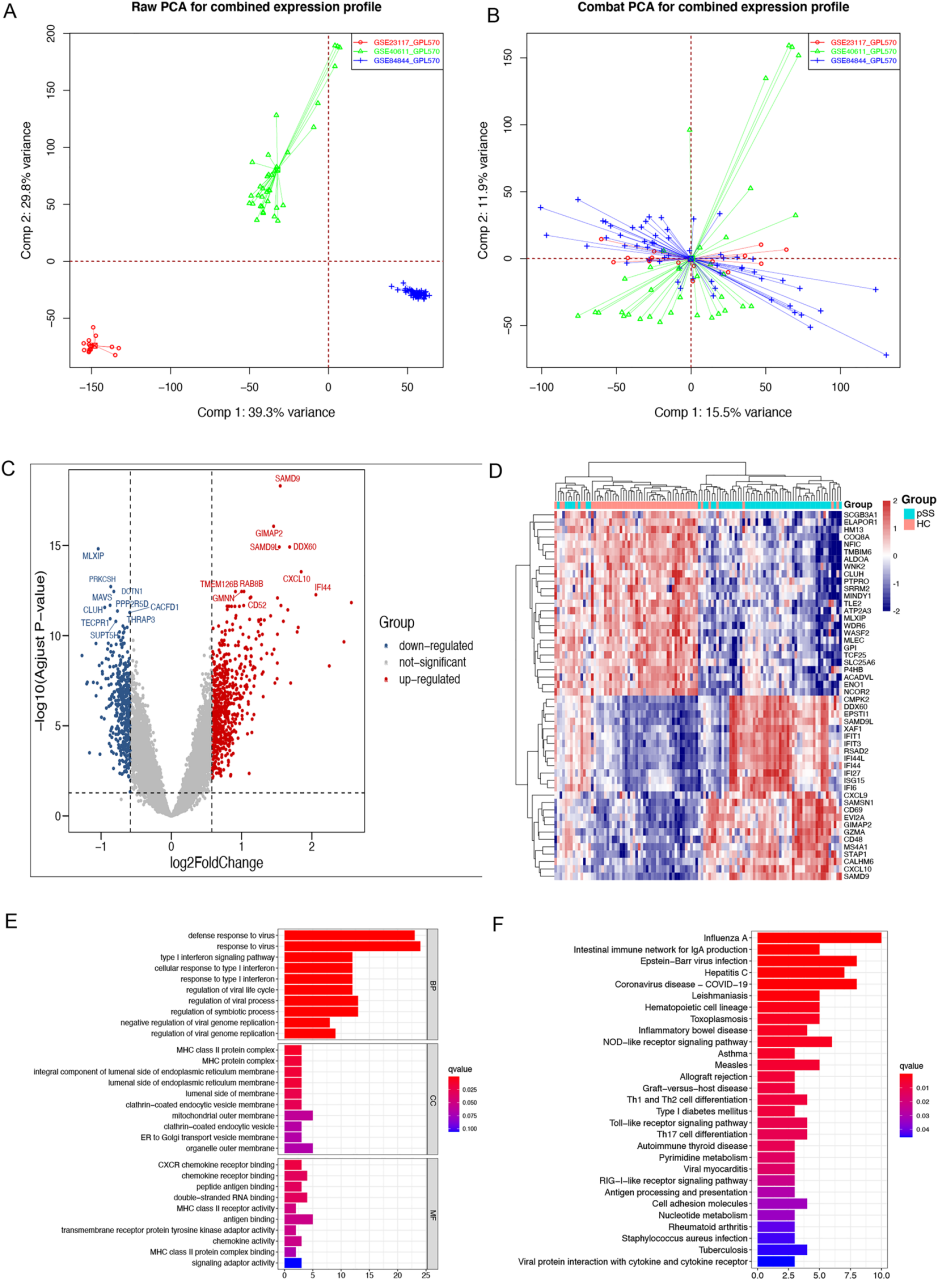
Analyses of DEGs in the training dataset. (**A**, **B**) Distribution and PCA before and after removing the batch effect. (**C**) Volcano plot of DEGs. (**D**) Heatmap of the 50 DEGs. (**E**) GO function enrichment analysis of the DEGs. (**F**) KEGG enrichment analysis of the DEGs.

**Figure 3:**
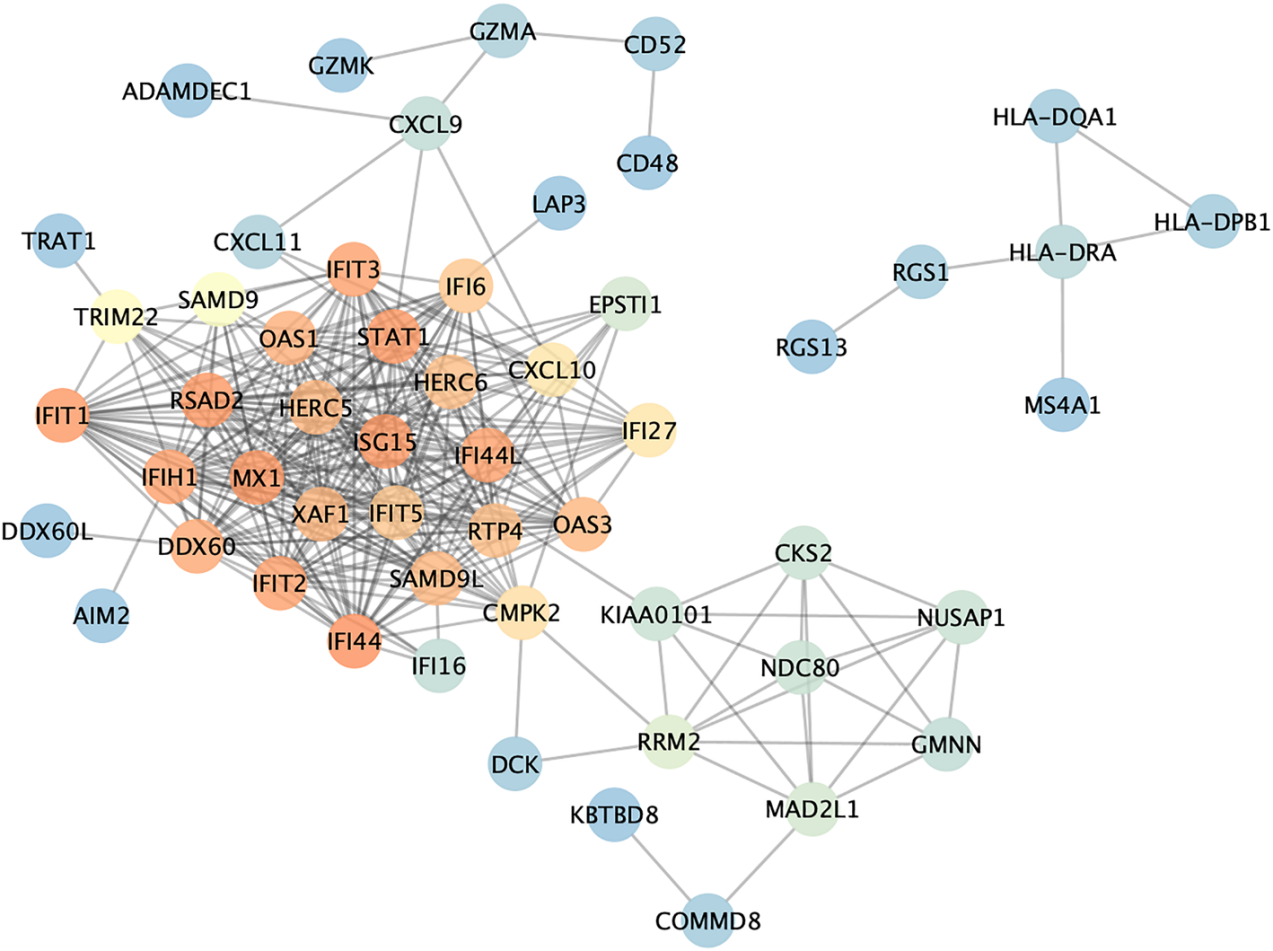
A network view of the pSS PPI network. Color is used to show the degree, with yellower genes indicating a higher degree, and bluer genes indicating a lower degree.

We combined the three datasets (GSE23117, GSE40611, and GSE84844) into a training cohort. The batch effect was mitigated after applying the ‘ComBat’ algorithm (Fig. 2A,B). In total, 96 DEGs were found between the pSS and normal samples using the “limma” package, of which 85 were upregulated (*SAMD9, GIMAP2,* and *DDX60*, among many others) and 11 were downregulated (for example, *MLXIP, WASF2,* and *NFIC*). Supplementary Table 1 presents the list of DEGs. Gene heatmaps (Fig. 2C) and volcano maps (Fig. 2D) were used to represent the DEG distributions. As a result of the GO functional classification, DEGs were mostly enriched in defense response to virus and the type I interferon signaling pathways, and in cellular response to type I interferon. KEGG functional analysis revealed that 96 DEGs were associated with the intestinal immune network for IgA production and the NOD-like receptor signaling pathway (Fig. 2E,F). Using STRING online database analysis of the PPI network, we obtained 400 pairs of proteins (96 proteins in total). Pairs with a combined score of more than 0.6 were visualized using the ‘Cytoscape’ software. Generally, the higher the degree of a node, the more important it is. CXCL10, NDC80, ISG15, SAMD9L, and HERC5 were identified as hub genes of the network. (Fig. 3).

### Random forest screening for signature genes

**Figure 4:**
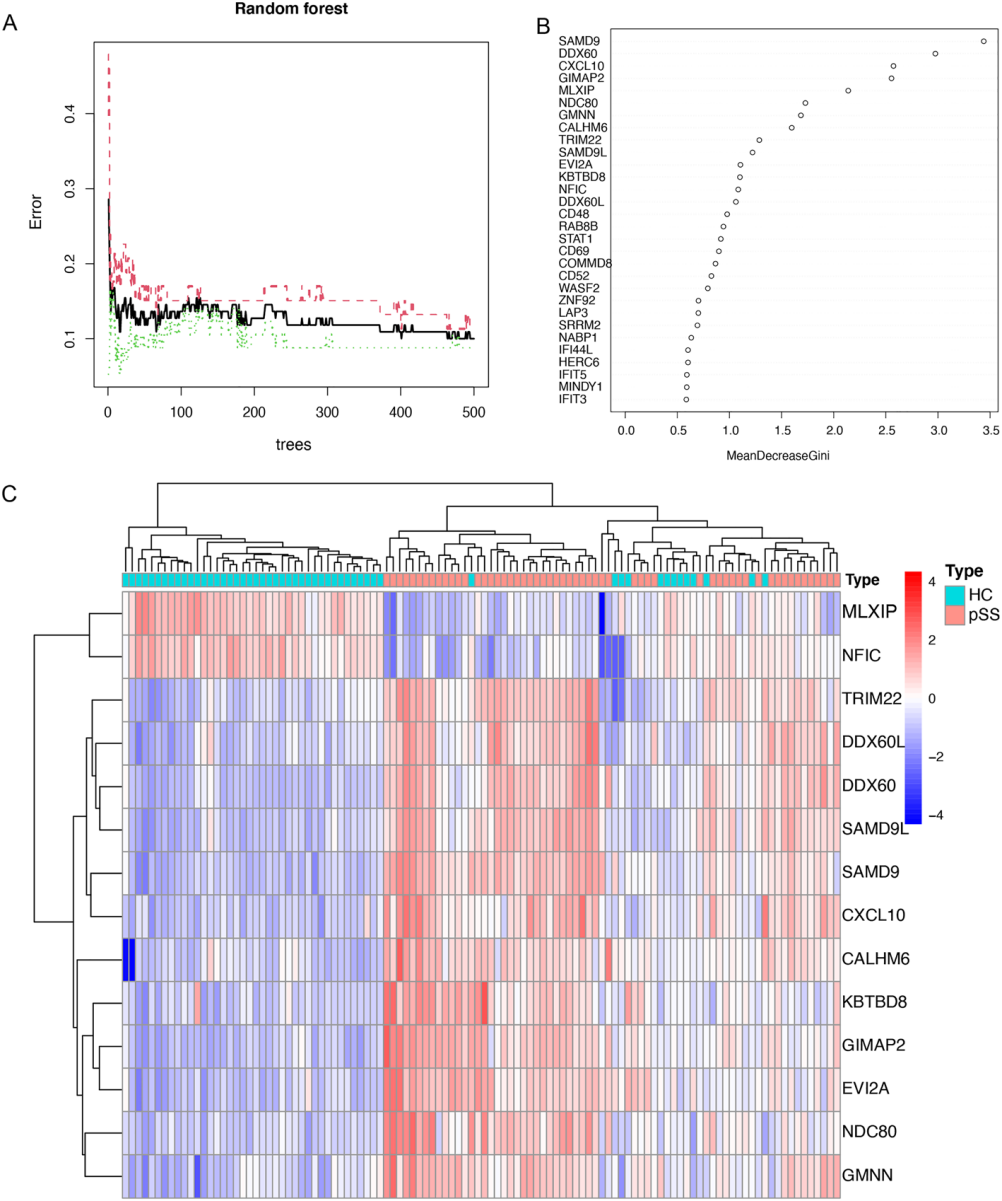
Random Forest analysis. (**A**) Correlation plot between RF trees and model error. (**B**) Gini coefficients were used in the RF classifiers to provide the following results. The importance index is on the x-axis, and the genetic variable is on the y-axis. (**C**) The heatmap of fourteen key genes generated by RF.

To obtain more reliable pSS signature genes, 96 DEGs were input into the RF classifier. For the 1 to 96 variables, a recurrent RF classification was carried out and used to calculate the average error rate of the model. Ultimately, the model with 401 trees was selected as the final parameter by analyzing the relationship between the model error and the number of decision trees (Fig. 4A). The relative importance of each genus was determined based on MeanDecreaseGini (Fig. 4B). We selected 14 DEGs with MeanDecreaseGini > 1 as the pSS-signature genes for ANN analysis, 12 of which (*SAMD9, DDX60, CXCL10, GIMAP2, NDC80, GMNN, CALHM6, TRIM22, SAMD9L, EVI2A, KBTBD8,* and *DDX60L*) were upregulated and two of which (*MLXIP* and *NFIC*) were downregulated. Figure 4B shows that among the twelve variables, SAMD9 and DDX60 were the most important, followed by CXCL10, GIMAP2, MLXIP, and NDC80. The heat plot (Fig. 4C) showed that the activity of 14 pSS signature genes could distinguish pSS samples from normal samples.

### Construction and validation of the Machine Learning model

**Figure 5:**
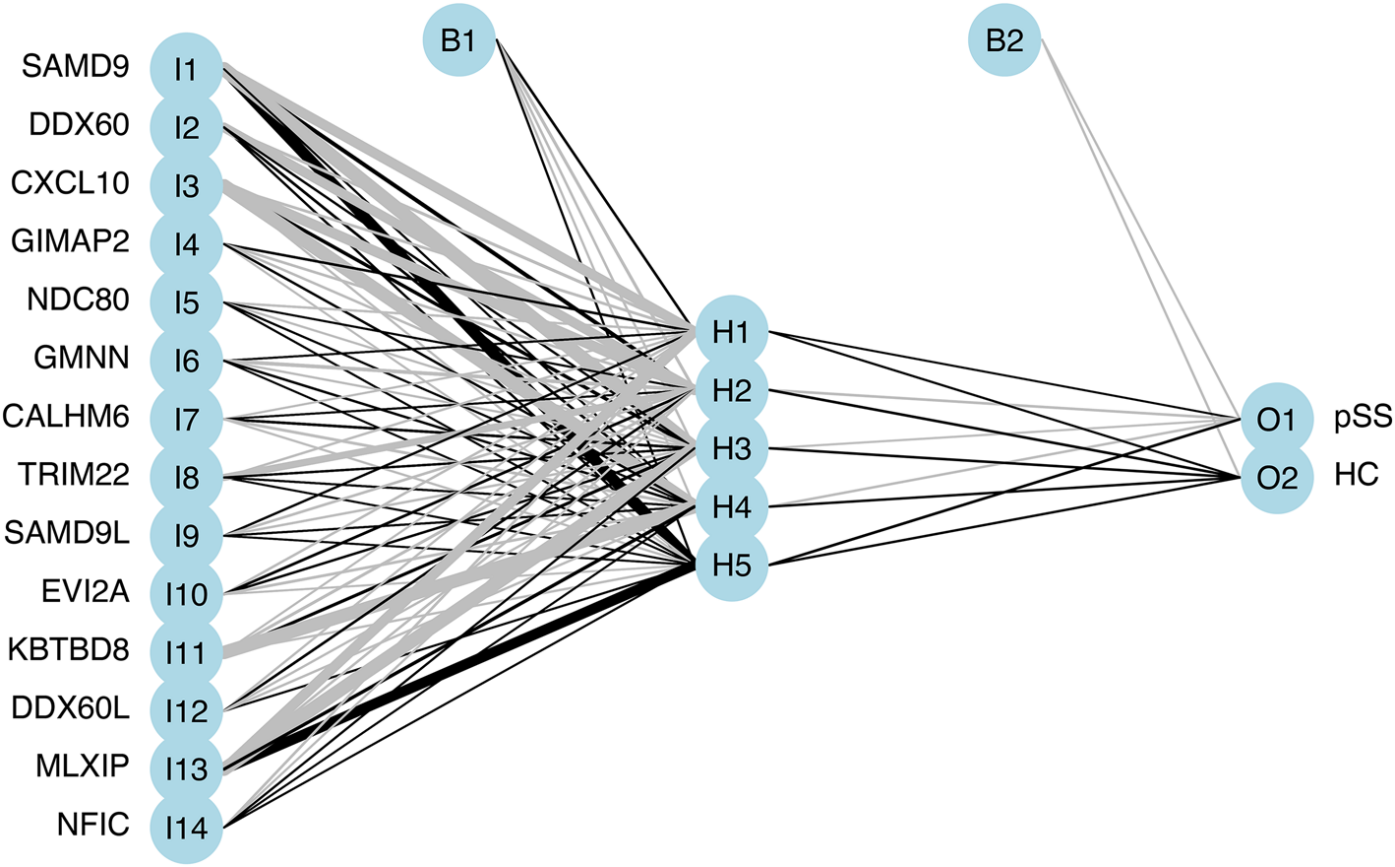
Results of artificial neural networks visualized.

**Figure 6:**
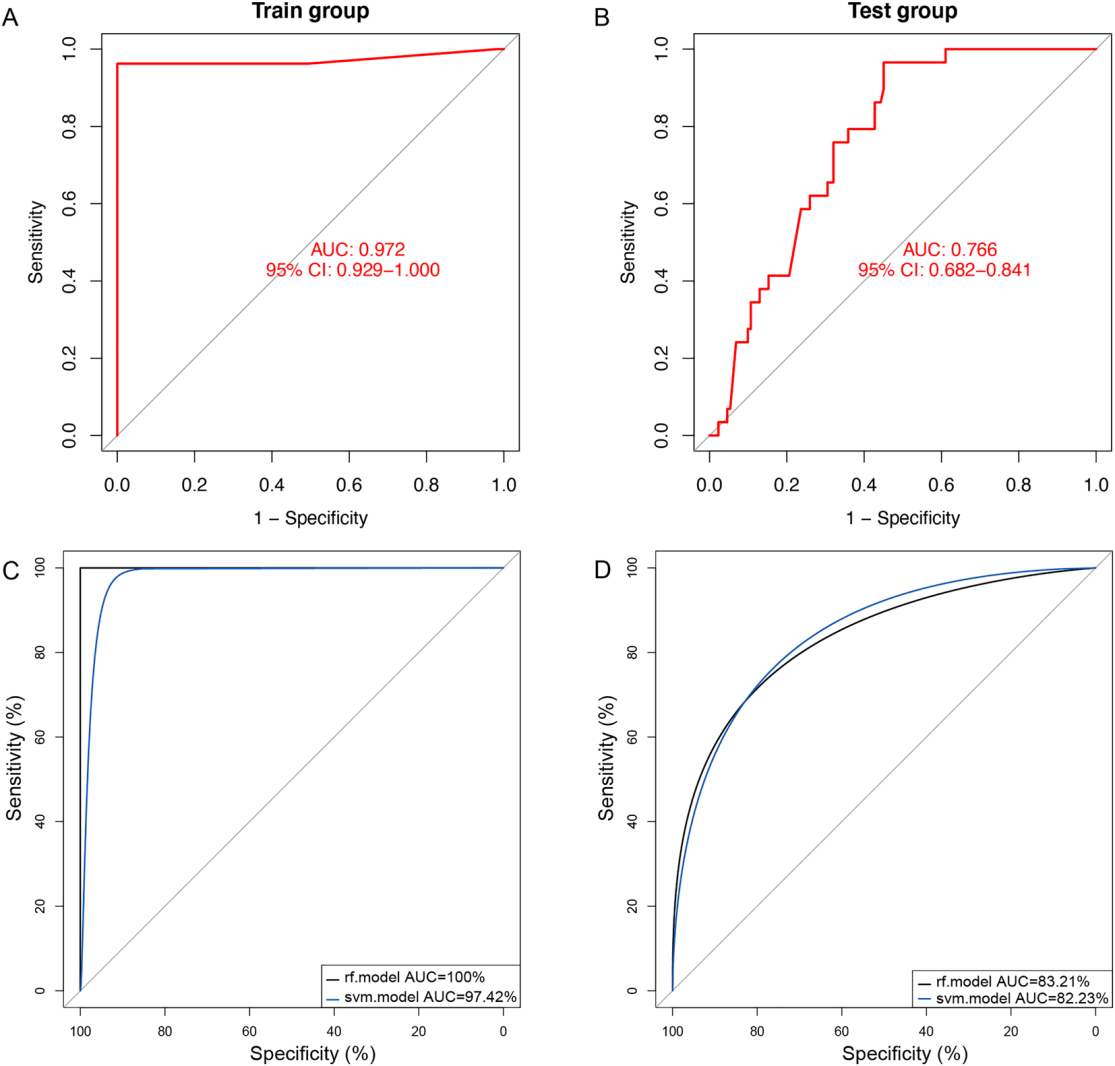
Evaluation of training and validation datasets using ROC curves and their AUC values. (**A**) ROC curve of ANN in training set. (**B**) ROC curve of ANN in testing set. (**C**) ROC curve of RF and SVM in training set. (**D**) ROC curve of RF and SVM in testing set.

The diagnostic model we developed for pSS was based on three machine learning algorithms. First, we converted the 14 pSS-signature genes expression into ‘Gene Score’ in order to perform an ANN analysis. The ANN consisted of three layers (input, hidden, and output). The number of nodes in the input and output layers were 14 (number of input signature genes) and two (pSS or HC (non-pSS)), respectively (Fig. 5). The pSS-specific scoring model was formulated using the expression ‘GeneExpression’ × ‘NeuralNetworkWeight’. The area under the ROC curve was used to measure performance. In the training dataset, the AUC was 0.972, accuracy was 0.9812, precision was 1.00, recall was 0.9661, and F1-score was 0.9828 (Fig. 6A and Supplementary Table 2). In the test dataset, the AUC was 0.766, accuracy was 0.7714, precision was 0.9277, recall was 0.5878, and F1-score was 0.7196 (Fig. 6B and Supplementary Table 3).

The results of the study indicate that in the training set, the RF model achieved perfect scores (values = 1) for AUC, accuracy, precision, recall, and F1-score, while the Support Vector Machine (SVM) model achieved a slightly lower AUC score of 0.9742, with accuracy, precision, recall, and F1-score values of 0.9455, 0.9322, 0.9649, and 0.9483, respectively (Fig. 6C and Supplementary Tables 4 and 5). In the testing set, the RF model achieved an AUC score of 0.8321, with accuracay, precision, recall, and F1-score values of 0.8188, 0.8188, 1.00, and 0.9003, respectively. Similarly, the SVM model achieved an AUC score of 0.8223, with accuracy, precision, recall, and F1-score values of 0.8188, 0.8188, 1.00, and 0.9003, respectively (Fig. 6D and Supplementary Tables 6 and 7). The results indicated that this model may discriminate effectively between pSS and non-pSS samples. It was the RF model that produced the best prediction performance out of the three models tested. In the end, we constructed a diagnostic model based on 14 genes using RF.

### Immune cell infiltration analysis

**Figure 7:**
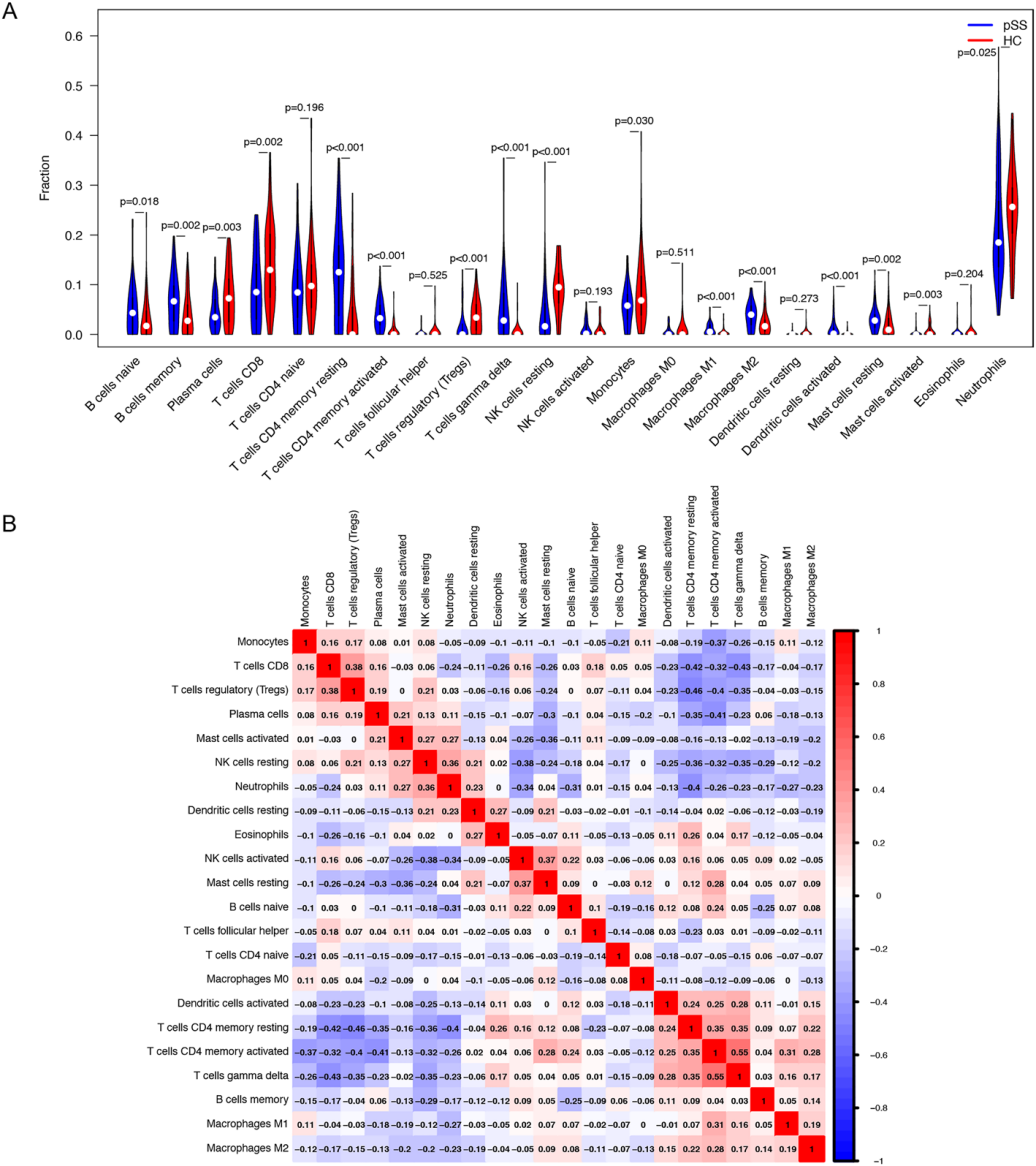
A review of the immunological landscape of pSS. (**A**) Twenty-two immune-cell subtypes were compared between the HC and pSS groups. (**B**) Correlation analysis of infiltrating immune cells.

We used CIBERSORT to analyze 22 immune cell phenotypes in the training set to determine whether they were associated with the pSS and non-pSS groups and with immune infiltration. The following phenotypes were found to be relatively abundant in pSS: naïve and memory B cells; CD4 memory resting, CD4 memory activated, and γδ T cells; M0 and M2 macrophages; dendritic cells; and both activated and resting mast cells. Meanwhile, in HC, the following phenotypes were relatively abundant: plasma cells; CD8 and regulatory (Tregs) T cells; resting NK cells; monocytes; mast cells; and neutrophils (Fig. 7A). The measured correlation for immune cell infiltration is shown in Fig. 7B.

## Discussion

Currently, pSS is diagnosed based on functional (Schirmer’s test), serological (anti-Ro/SSA), and histological (labial minor salivary gland or salivary gland) tests^27,28^. However, due to a combination of the heterogeneity of the disease, its complex clinical phenotypes, and the lack of effective biomarkers for early screening, most patients are diagnosed with an advanced form of the disease on presentation. Thus, it is crucial to develop effective screening tools and assess risk factors early.

We obtained four datasets (GSE23117, GSE40611, GSE84844, and GSE66795) from the GEO in order to build and validate a diagnostic model for pSS. We identified 96 genes that are expressed differently between the pSS and HC groups; enrichment analysis indicated that these DEGs were mostly involved in immunological processes. The ‘defense response to viruses’ and ‘type I interferon signaling pathway’ were the most enriched GO terms. These results are consistent with previous studies that have shown a relationship between interferon signaling and pSS. Titers of anti-Ro and anti-La autoantibodies are positively associated with type I interferon overexpression genes even in pSS^29,30^. Type I interferons are important components of the innate immune system that facilitate inhibition of viral infections via adaptive immunity^31^. The intestinal immune network governing IgA production was observed to be the most enriched KEGG pathway in the pSS group. In normal physiology, host-gut microbiota interactions are complex and multifaceted. Exposure to gut microbes stimulates continuous diversification of B-cell repertoires and constant production of IgA antibodies, both T-dependent and T-independent^32^. Our analysis of GO and KEGG pathways revealed that these differentially expressed proteins could be involved in the development of pSS.

Fourteen DEGs were identified by RF analysis: *SAMD9, DDX60, CXCL10, GIMAP2, NDC80, GMNN, CALHM6, TRIM22, SAMD9L, EVI2A, KBTBD8, DDX60L, MLXIP,* and *NFIC.* Our findings are consistent with those of previous studies. AMDS9 is a genetically regulated anti-inflammatory factor in patients with rheumatoid arthritis^33^.

It is estimated that DDX60L and DDX60 share 70% of their amino acid sequences^34^. The *DDX60L* gene is activated by interferons. In the innate immune system, *DDX60L* proteins recognize viral RNA molecules in order to protect against viral infections^35^. So far, there is little information available about the function of the DDX60L. It has been shown that DDX60L is associated with HIV host factors^36^, and childhood obesity^37^. This gene encodes a component of the NDC80 kinetochore complex, which is responsible for organizing and stabilizing interactions between microtubules and keratochromas^38^. The GMNN gene regulates the cell cycle. By inhibiting DNA replication licensing and histone H4 acetylation, GMNN promotes cell proliferation^39^. It is thought that CALHM6 regulates infection-related immunity^40^. Apart from pSS, a number of other autoimmune diseases are thought to be influenced by CXCL10, which recruits immune cells to sites of inflammation^41^. The GIMAP family of proteins regulates lymphocyte apoptosis by acting as GTPases of immunity-associated proteins^42^. In lymphocytes, GIMAP2 heterodimerizes with the GIMAP7 protein to activate GIMAP7 function^43,44^. According to these studies, multiple GIMAP proteins contribute to the survival of T cells. Approximately 70% of pSS patients who meet the diagnostic criteria have serum autoantibodies against several intracellular proteins (e.g., TRIM21 (Ro52), La/SSB)^45,46^. Ro52/TRIM21 plays a crucial role in antibody-dependent pathogen neutralization^47^. A tumor suppressor, SAMD9L is repressed by the p53 pathway in breast and hepatocellular tissues^48^. In hematopoietic tissue, SAMD9L plays a crucial role in regulating cell proliferation^49^. It is possible that Evi2a is a lymphocyte-specific tumor suppressor, which could play a role in BCR activation^50^. BBK protein that has been identified as being found in the Golgi apparatus and translocating to the forming spindle after KBTBD8 is the first entry into mitosis^51^. The findings presented here indicate that KBTBD8 is also essential for the healthy function of ovarian epithelium^52^. The MLXIP interacts with Max-like protein X (MLX) to activate transcription. Ovarian cancer cells migrate towards MLXLP, which was associated with a poor prognosis^53^. In mice, NFIC regulates the expression of PTEN/SENP8 and inhibits rheumatoid arthritis-induced inflammation^54^. Many of the variations have not yet been reported as being linked to pSS but have strong associations with other autoimmune disorders. A deeper understanding of the complex role these genes play in pSS requires further research.

We developed a diagnostic prediction model for patients with pSS utilizing machine learning algorithms, namely ANN, RF, and SVM, based on 14 genes. The diagnostic models for pSS using the aforementioned algorithms were successfully designed and achieved AUCs of 0.972, 1.00, and 0.9742 in the training and testing datasets, respectively. However, the AUCs for the validation set were 0.766, 0.8321, and 0.8223. The prediction properties of our model were deemed satisfactory. Nevertheless, the sample size of our cohort was limited, and further studies with larger-scale cohorts are required to validate our findings.

In addition, we examined the immune microenvironment of pSS. Multiple studies have shown that B cells are associated with disease activity in pSS^55^, while CD4 + T cells in pSS undergo premature aging due to lymphopenia^56^. A significant increase in dendritic cells has been observed in patients with pSS, which is closely related to Type I interferons^29^; overexpression has also been observed in mast cells, which produce transforming growth factor β1 and promote tissue fibrosis^57^. Conversely, a major reduction in NKT-like cells has been observed in pSS, which may be contributing to the pathogenesis of the disease^58^. Researchers may be able to identify novel immunotherapies for pSS by further studying the host immune response.

This study has several limitations. First, for further validation of the diagnostic model, large cohorts are needed. Second, the predictive performance of the different pSS diagnostic model needs to be validated in larger cohort.

Here, we proposed and externally verified a pSS diagnostic model. Our model is both specific and sensitive and shows great potential as a basis for the development of new diagnostic tools for pSS. We also explored the immune status of pSS, and our data provide the impetus for further analyses in order to gain a deeper understanding of the condition. Further research into the possible applications of our model in clinical settings is needed in order to improve patient outcomes.

## Supplementary Information

Supplementary Tables.

## Data Availability

The datasets generated during the current study are available in the GEO database (http://www.ncbi.nlm.nih.gov/geo/) with the accession no GSE23117, GSE40611, GSE84844, and GSE66795.
