# Supplementary material for: Development and verification of a combined diagnostic model for primary Sjögren's syndrome by integrated bioinformatics analysis and machine learning
Source: Sci Rep. 2023 May 27;13:8641. doi: 10.1038/s41598-023-35864-4 (PMC10224947; doi:10.1038/s41598-023-35864-4)
Supplement: Supplementary file 1 — Supplementary Tables. [file 41598_2023_35864_MOESM1_ESM.docx]

Supplement Table 1 Genes with significantly different expression between pSS and HC

| gene_symbol | logFC | AveExpr | t | P.Value | adj.P.Val | B | group |
| --- | --- | --- | --- | --- | --- | --- | --- |
| SAMD9 | 1.550646076 | 6.772188954 | 12.47444578 | 3.81E-23 | 5.00E-19 | 41.83521683 | up |
| GIMAP2 | 1.456218912 | 7.596716265 | 11.38784573 | 1.32E-20 | 8.67E-17 | 36.17537988 | up |
| DDX60 | 1.684090759 | 7.239803674 | 10.79011538 | 3.36E-19 | 1.22E-15 | 33.03796942 | up |
| SAMD9L | 1.537145678 | 7.410535287 | 10.77151188 | 3.71E-19 | 1.22E-15 | 32.94017724 | up |
| MLXIP | -1.041037941 | 7.960781348 | -10.68794293 | 5.84E-19 | 1.54E-15 | 32.50082091 | down |
| CXCL10 | 1.848585649 | 5.652604657 | 10.11410755 | 1.31E-17 | 2.87E-14 | 29.48321896 | up |
| RAB8B | 1.034349471 | 8.701774481 | 9.555621145 | 2.69E-16 | 3.58E-13 | 26.55196124 | up |
| GMNN | 1.000187897 | 5.329580877 | 9.553175741 | 2.72E-16 | 3.58E-13 | 26.53915353 | up |
| IFI44 | 2.058351378 | 7.23384146 | 9.441555415 | 4.97E-16 | 5.44E-13 | 25.95488782 | up |
| CD52 | 1.133291198 | 7.518947192 | 9.370347698 | 7.29E-16 | 7.37E-13 | 25.58253331 | up |
| NDC80 | 1.1203938 | 3.859937905 | 9.337351945 | 8.71E-16 | 8.15E-13 | 25.41010149 | up |
| EVI2A | 1.511308128 | 8.109195793 | 9.325015924 | 9.30E-16 | 8.15E-13 | 25.34565313 | up |
| IFI44L | 2.563362761 | 7.445046706 | 9.20219937 | 1.80E-15 | 1.48E-12 | 24.70458118 | up |
| EPSTI1 | 1.560927321 | 7.506268588 | 9.109838037 | 2.96E-15 | 2.15E-12 | 24.22321433 | up |
| DDX60L | 1.030189552 | 8.078289014 | 9.100878453 | 3.10E-15 | 2.15E-12 | 24.17655498 | up |
| CD69 | 1.65821352 | 5.585414541 | 8.931664681 | 7.68E-15 | 3.74E-12 | 23.2966243 | up |
| GZMA | 1.413810392 | 6.970660761 | 8.763192168 | 1.89E-14 | 8.00E-12 | 22.423232 | up |
| BIRC3 | 1.114246595 | 7.434605112 | 8.66106319 | 3.25E-14 | 1.26E-11 | 21.89523365 | up |
| CKS2 | 1.273454114 | 4.896225638 | 8.653180737 | 3.39E-14 | 1.27E-11 | 21.85453086 | up |
| CD48 | 1.323998048 | 8.72164952 | 8.633149949 | 3.77E-14 | 1.38E-11 | 21.7511299 | up |
| RTP4 | 1.241736267 | 6.149528333 | 8.628007428 | 3.88E-14 | 1.38E-11 | 21.72459125 | up |
| IFIT3 | 1.59496678 | 8.955388002 | 8.59401088 | 4.65E-14 | 1.61E-11 | 21.54922649 | up |
| TRIM22 | 1.2445084 | 9.529716259 | 8.582559603 | 4.94E-14 | 1.62E-11 | 21.49018843 | up |
| STAT1 | 1.272965535 | 10.0488542 | 8.497866842 | 7.74E-14 | 2.36E-11 | 21.05404973 | up |
| CMPK2 | 1.80660857 | 8.201677966 | 8.402546159 | 1.28E-13 | 3.51E-11 | 20.56428388 | up |
| IFIT2 | 1.235373262 | 8.524838063 | 8.27624295 | 2.50E-13 | 6.08E-11 | 19.91724527 | up |
| IFIT1 | 1.791590032 | 8.378356585 | 8.263538701 | 2.67E-13 | 6.26E-11 | 19.85228952 | up |
| CALHM6 | 1.510776179 | 7.278136547 | 8.194492602 | 3.84E-13 | 8.41E-11 | 19.49968488 | up |
| MS4A4A | 1.296872476 | 5.195994728 | 8.187147459 | 3.99E-13 | 8.47E-11 | 19.46221739 | up |
| STAMBPL1 | 1.019549525 | 6.550463166 | 8.186775583 | 4.00E-13 | 8.47E-11 | 19.46032068 | up |
| IFIH1 | 1.25337177 | 6.972901229 | 8.149299098 | 4.87E-13 | 9.99E-11 | 19.26928636 | up |
| NABP1 | 1.102459774 | 7.088268044 | 8.103993279 | 6.17E-13 | 1.23E-10 | 19.03863906 | up |
| HERC6 | 1.206199283 | 6.708535887 | 8.057107411 | 7.89E-13 | 1.53E-10 | 18.80029804 | up |
| MS4A1 | 2.458060559 | 8.570849591 | 7.964477256 | 1.28E-12 | 2.22E-10 | 18.33050171 | up |
| WASF2 | -1.072425121 | 8.298226679 | -7.911571869 | 1.69E-12 | 2.73E-10 | 18.06284661 | down |
| BCL2A1 | 1.285519556 | 5.772700033 | 7.868272937 | 2.12E-12 | 3.19E-10 | 17.84416491 | up |
| CLEC2B | 1.064611639 | 6.162066299 | 7.787326578 | 3.22E-12 | 4.41E-10 | 17.43627298 | up |
| MCOLN2 | 1.048656765 | 6.091736614 | 7.76201951 | 3.67E-12 | 4.92E-10 | 17.30900421 | up |
| AIM2 | 1.133856368 | 5.991028597 | 7.747139529 | 3.97E-12 | 5.21E-10 | 17.23423082 | up |
| KBTBD8 | 1.083473518 | 5.184989205 | 7.722182036 | 4.51E-12 | 5.79E-10 | 17.10891397 | up |
| SAMSN1 | 1.46257133 | 6.697271259 | 7.721253022 | 4.54E-12 | 5.79E-10 | 17.10425156 | up |
| STAP1 | 1.358975281 | 5.484102539 | 7.60692411 | 8.19E-12 | 9.69E-10 | 16.53179729 | up |
| XAF1 | 1.434521899 | 8.981578781 | 7.582967864 | 9.26E-12 | 1.07E-09 | 16.41218686 | up |
| NFIC | -1.272006905 | 7.877950877 | -7.538341106 | 1.17E-11 | 1.28E-09 | 16.18969495 | down |
| KMO | 1.135270582 | 4.796228755 | 7.521173838 | 1.27E-11 | 1.35E-09 | 16.10421922 | up |
| LAP3 | 1.061543363 | 8.879421756 | 7.509689566 | 1.35E-11 | 1.42E-09 | 16.04707476 | up |
| GPR65 | 1.138521162 | 6.601543328 | 7.504922382 | 1.38E-11 | 1.44E-09 | 16.02336223 | up |
| IFIT5 | 1.060809718 | 7.430153971 | 7.446228664 | 1.87E-11 | 1.87E-09 | 15.73182318 | up |
| RGS1 | 1.279854977 | 5.141811383 | 7.43492179 | 1.98E-11 | 1.94E-09 | 15.67574873 | up |
| NUSAP1 | 1.121955965 | 5.226213345 | 7.34887863 | 3.07E-11 | 2.80E-09 | 15.24998839 | up |
| CXCL9 | 1.509107096 | 5.249063432 | 7.302873921 | 3.88E-11 | 3.34E-09 | 15.02305487 | up |
| PCLAF | 1.127526158 | 5.554586062 | 7.24613064 | 5.18E-11 | 4.20E-09 | 14.74384803 | up |
| SRRM2 | -1.026211833 | 7.83174375 | -7.225005495 | 5.77E-11 | 4.51E-09 | 14.64010172 | down |
| IFI27 | 2.249527893 | 6.220147658 | 7.207204163 | 6.31E-11 | 4.82E-09 | 14.5527644 | up |
| GPR18 | 1.170233083 | 6.408593046 | 7.182915352 | 7.14E-11 | 5.30E-09 | 14.43372552 | up |
| RPL22L1 | 1.046781906 | 6.104611647 | 7.175153081 | 7.42E-11 | 5.42E-09 | 14.39571406 | up |
| GZMK | 1.190996256 | 7.186718084 | 7.149845218 | 8.44E-11 | 6.09E-09 | 14.27188861 | up |
| MAD2L1 | 1.154666449 | 4.495568185 | 7.097812804 | 1.10E-10 | 7.55E-09 | 14.0178216 | up |
| DCK | 1.029323618 | 7.357689123 | 7.047133719 | 1.42E-10 | 9.21E-09 | 13.77104127 | up |
| LAMP3 | 1.307512694 | 6.160756633 | 7.015433555 | 1.66E-10 | 1.04E-08 | 13.61702496 | up |
| HERC5 | 1.291383535 | 7.568340521 | 6.943111407 | 2.38E-10 | 1.34E-08 | 13.26666525 | up |
| ITGA4 | 1.006550919 | 7.405948674 | 6.923424974 | 2.63E-10 | 1.42E-08 | 13.1715453 | up |
| TFEC | 1.189712276 | 4.913005842 | 6.878523303 | 3.29E-10 | 1.73E-08 | 12.95499855 | up |
| TNFRSF17 | 1.071863746 | 5.577962223 | 6.868137484 | 3.46E-10 | 1.80E-08 | 12.90499235 | up |
| TRAT1 | 1.18393274 | 5.054707548 | 6.803018311 | 4.79E-10 | 2.29E-08 | 12.59216082 | up |
| ISG15 | 1.505897822 | 8.319528151 | 6.781707881 | 5.32E-10 | 2.46E-08 | 12.49005427 | up |
| ST8SIA4 | 1.112868867 | 6.877854557 | 6.733483256 | 6.75E-10 | 2.98E-08 | 12.25948853 | up |
| PTPRO | -1.19493474 | 9.73610165 | -6.673898695 | 9.05E-10 | 3.84E-08 | 11.97557813 | down |
| MINDY1 | -1.110307955 | 9.22397572 | -6.669094718 | 9.27E-10 | 3.90E-08 | 11.95273527 | down |
| RSAD2 | 1.699166101 | 7.458870542 | 6.644870669 | 1.04E-09 | 4.26E-08 | 11.8376588 | up |
| IFI6 | 1.474415917 | 8.549358036 | 6.644803956 | 1.04E-09 | 4.26E-08 | 11.83734213 | up |
| P4HB | -1.053973927 | 9.602313792 | -6.643124663 | 1.05E-09 | 4.28E-08 | 11.82937142 | down |
| ALDOA | -1.204895079 | 10.32364525 | -6.616315814 | 1.20E-09 | 4.68E-08 | 11.70224337 | down |
| IFI16 | 1.072014334 | 9.345192292 | 6.580676369 | 1.43E-09 | 5.34E-08 | 11.53359069 | up |
| OAS1 | 1.175120609 | 7.690201041 | 6.534569476 | 1.79E-09 | 6.36E-08 | 11.31600505 | up |
| COMMD8 | 1.047779353 | 7.425858006 | 6.533602475 | 1.80E-09 | 6.38E-08 | 11.31144895 | up |
| ZC3H12D | 1.079230069 | 6.945050429 | 6.51087224 | 2.01E-09 | 6.99E-08 | 11.20444123 | up |
| CXCL11 | 1.068638076 | 3.38292845 | 6.441830228 | 2.81E-09 | 9.17E-08 | 10.88045091 | up |
| BANK1 | 1.12927958 | 6.929122933 | 6.309425597 | 5.33E-09 | 1.53E-07 | 10.26361975 | up |
| S100A8 | 1.031998504 | 8.673922604 | 6.308719733 | 5.35E-09 | 1.53E-07 | 10.26034758 | up |
| TMBIM6 | -1.070857173 | 10.78674209 | -6.21882475 | 8.22E-09 | 2.17E-07 | 9.84506378 | down |
| HLA-DRA | 1.308310987 | 10.07803374 | 6.185729668 | 9.63E-09 | 2.42E-07 | 9.692906994 | up |
| SNX10 | 1.011468438 | 7.505889205 | 6.166643402 | 1.05E-08 | 2.62E-07 | 9.605338396 | up |
| RGS13 | 1.095730632 | 3.119416145 | 6.017099753 | 2.14E-08 | 4.63E-07 | 8.92393168 | up |
| FCRL2 | 1.097378596 | 5.925545306 | 5.9383212 | 3.09E-08 | 6.30E-07 | 8.568419653 | up |
| RRM2 | 1.011027885 | 5.503262616 | 5.720629331 | 8.47E-08 | 1.44E-06 | 7.598993114 | up |
| SNRPG | 1.081902263 | 7.729250314 | 5.62215544 | 1.33E-07 | 2.06E-06 | 7.166987647 | up |
| ZNF92 | 1.175865416 | 6.122213256 | 5.581231899 | 1.60E-07 | 2.40E-06 | 6.988693903 | up |
| OAS3 | 1.006443089 | 6.779308869 | 5.546627229 | 1.87E-07 | 2.74E-06 | 6.838506952 | up |
| HLA-DPB1 | 1.009850526 | 10.03115992 | 5.430196855 | 3.15E-07 | 4.19E-06 | 6.337145842 | up |
| MX1 | 1.150862845 | 8.507505987 | 5.423062745 | 3.26E-07 | 4.30E-06 | 6.306626971 | up |
| ADAMDEC1 | 1.146533051 | 3.976845232 | 4.9424436 | 2.63E-06 | 2.36E-05 | 4.307261569 | up |
| ATP5F1C | 1.084055051 | 9.158819458 | 4.890654333 | 3.28E-06 | 2.81E-05 | 4.098815903 | up |
| ELAPOR1 | -1.165177637 | 8.162281971 | -4.160641167 | 6.13E-05 | 0.000314468 | 1.321689021 | down |
| SCGB3A1 | -1.015730633 | 6.460162396 | -4.100014081 | 7.71E-05 | 0.000378226 | 1.105498315 | down |
| HLA-DQA1 | 1.108279292 | 5.862149683 | 3.274472213 | 0.001396968 | 0.004388611 | -1.591118934 | up |

Supplement Table 2 The confusion matrix of training dataset by ANN.

| Group | pSS | HC |
| --- | --- | --- |
| pSS | 57 | 2 |
| HC | 0 | 51 |

Supplement Table 3 The confusion matrix of testing dataset by ANN.

| Group | pSS | HC |
| --- | --- | --- |
| pSS | 77 | 54 |
| HC | 6 | 23 |

Supplement Table 4 The confusion matrix of training dataset by RF.

| Group | pSS | HC |
| --- | --- | --- |
| pSS | 57 | 0 |
| HC | 0 | 53 |

Supplement Table 5 The confusion matrix of training dataset by SVM.

| Group | pSS | HC |
| --- | --- | --- |
| pSS | 55 | 4 |
| HC | 2 | 49 |

Supplement Table 6 The confusion matrix of testing dataset by RF.

| Group | pSS | HC |
| --- | --- | --- |
| pSS | 131 | 29 |
| HC | 0 | 0 |

Supplement Table 7 The confusion matrix of testing dataset by SVM.

| Group | pSS | HC |
| --- | --- | --- |
| pSS | 131 | 29 |
| HC | 0 | 0 |
